# Biochar of Spent Coffee Grounds as Per Se and Impregnated with TiO_2_: Promising Waste-Derived Adsorbents for Balofloxacin

**DOI:** 10.3390/molecules26082295

**Published:** 2021-04-15

**Authors:** Marwa El-Azazy, Ahmed S. El-Shafie, Hagar Morsy

**Affiliations:** Department of Chemistry and Earth Sciences, College of Arts and Sciences, Qatar University, Doha 2713, Qatar; aelshafie@qu.edu.qa (A.S.E.-S.); hm1604839@student.qu.edu.qa (H.M.)

**Keywords:** balofloxacin, green adsorbent, wastewater, spent coffee biochar, TiO_2_-impregnated biochar, Plackett-Burman design

## Abstract

Biochars (BC) of spent coffee grounds, both pristine (SCBC) and impregnated with titanium oxide (TiO_2_@SCBC) were exploited as environmentally friendly and economical sorbents for the fluroquinolone antibiotic balofloxacin (BALX). Surface morphology, functional moieties, and thermal stabilities of both adsorbents were scrutinized using SEM, EDS, TEM, BET, FTIR, Raman, and TG/*d*T analyses. BET analysis indicated that the impregnation with TiO_2_ has increased the surface area (50.54 m^2^/g) and decreased the pore size and volume. Batch adsorption experiments were completed in lights of the experimental set-up of Plackett-Burman design (PBD). Two responses were maximized; the % removal (%R) and the adsorption capacity (*q_e_*, mg/g) as a function of four variables: pH, adsorbent dosage (AD), BALX concentration ([BALX]), and contact time (CT). %R of 68.34% and 91.78% were accomplished using the pristine and TiO_2_@SCBC, respectively. Equilibrium isotherms indicated that Freundlich model was of a perfect fit for adsorption of BALX onto both adsorbents. Maximum adsorption capacity (*q_max_*) of 142.55 mg/g for SCBC and 196.73 mg/g for the TiO_2_@SCBC. Kinetics of the adsorption process were best demonstrated using the pseudo-second order (PSO) model. The adsorption-desorption studies showed that both adsorbents could be restored with the adsorption efficiency being conserved up to 66.32% after the fifth cycles.

## 1. Introduction

Antimicrobials represent an enormous category of chemicals that is used mainly in the remediation of infectious diseases. Such a superfamily includes many other superfamilies of pharmaceutically active compounds, such as antibiotics, antifungals, antivirals, antiseptics, etc. Having undeniable benefits for human and animal health, their use is becoming part of the daily routine. Over the past few decades, antibiotics in specific, were among the most significant emergent pollutants. Being extensively used within therapeutic or veterinary rehearsals, the improper disposal of antibiotics and their partial metabolism after application have resulted in a crisis. Even present as traces, antibiotics in water would cause serious impacts on the ecosystem. The appearance of antibiotic–resistant bacterial strains is another aspect of the crisis [1,2,3,4,5].

Fluoroquinolones (FQs) are believed to be the fourth major category of antibiotics in terms of production and consumption. As per the US Food and Drug Administration (FDA) report of 2011, 277,439 kg of quinolones were wholesaled for human use in USA corresponding to 8.4% of all antibiotics being sold. For veterinary purposes, 136 tons of FQs were sold in 2012 in the European Union [6,7]. The ubiquity of FQs in the aquatic systems raises concerns about the efficiency of wastewater treatment plants and demands an understanding of their occurrence and fate [8].

**Scheme 1:**
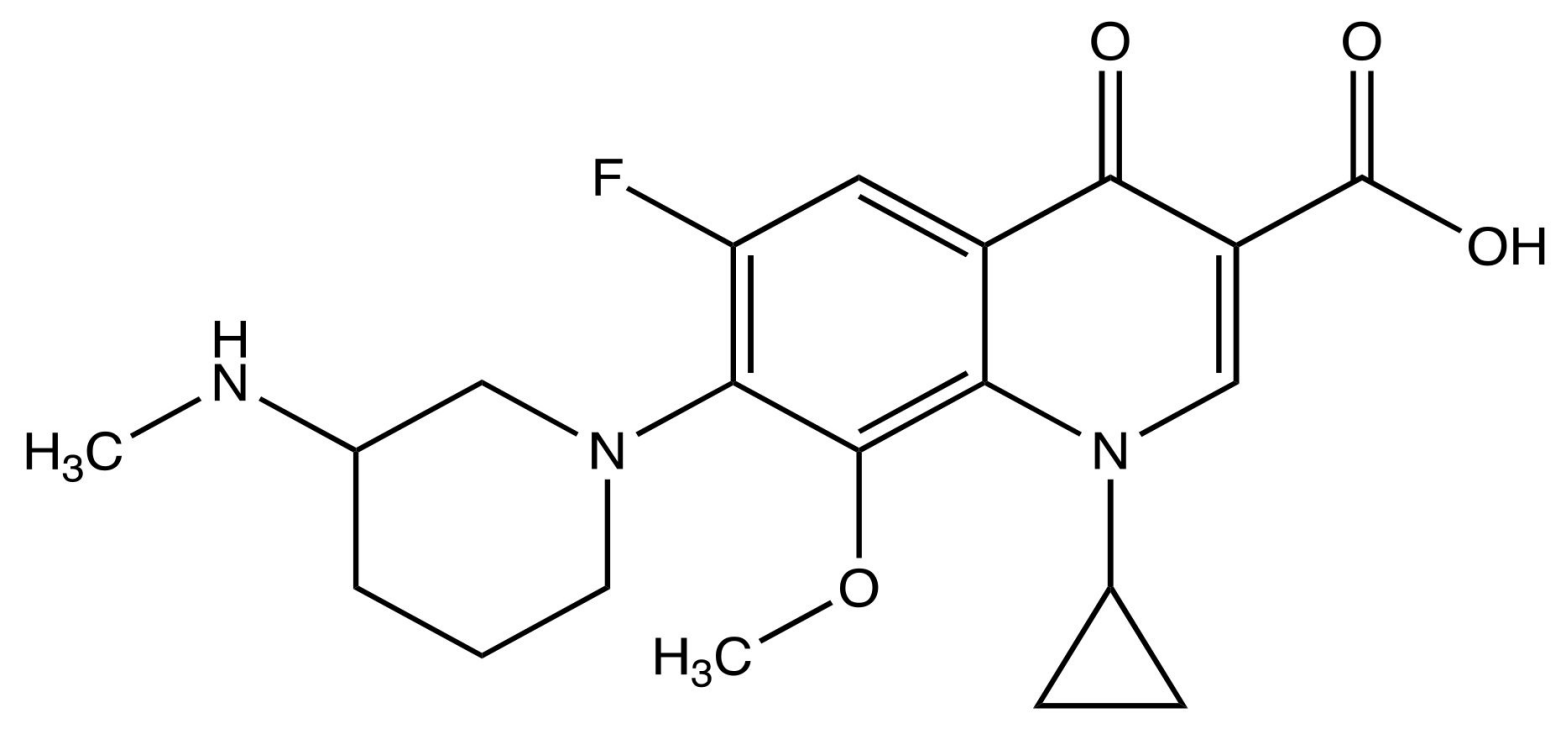
Chemical structure of BALX. Chemical Formula: C_20_H_24_FN_3_O_4_ (389.42 g/mol); IUPAC Name:1-Cyclopropyl-6-fluoro-8-methoxy-7-(3-methylaminopiperidin-1-yl)-4-oxoquinoline-3-carboxylic acid; pK_a_: 6.0 ± 0.2 [10].

Balofloxacin (BALX, Scheme 1) is a third generation FQ antibiotic. BALX is used mainly in the treatment of urinary tract infections, tonsilitis, pneumonia and its main route of excretion is renal. A literature survey reveals interesting studies on the occurrence, photochemistry, and toxicity of FQs in surface waters. While most of the investigations have targeted the elder members of the FQ family, fewer studies have been devoted to the new widely utilized BALX that has been detected in environmental waters. Interestingly, the photodegradation half-life of BALX was 58.0 min implying its fast decay under sunlight in surface water into toxic intermediates [9,10,11,12].

Offering irresistible advantages in terms of cost-efficiency, simplicity, high removal capability with a superior output quality, adsorption has been widely applied as a wastewater treatment approach for the removal of several pollutants [13,14,15] including FQs [16,17,18]. Yet, and to the best of our knowledge, no efforts have been made towards the removal of BALX using adsorption as an approach. Limitations such as cost and quick saturation of the adsorbent and hence an added cost for regeneration, hinder the applicability of adsorbents on the large scale. By and large, recycling of livestock and agricultural wastes into activated carbon overcomes these limitations. Moreover, the upcycling of these waste materials into value-added products helps to alleviate their hazardous impacts on the ecosystem.

Coffee is one of the most consumed beverages worldwide and till 1998 was the second most valued commodity exported by the developing countries after the crude oil. Recent statistics as per the International Coffee Organization (ICO) showed that the world coffee trade was around 10.21 million bags in January 2021 [18]. Spent coffee grounds (SCG) are the fine residues obtained after brewing powdered coffee. SCG is produced in huge amounts. According to the United States Department of Agriculture (USDA), an amount of around 650 kg of coffee residues could result from 1 ton of coffee. Therefore, the coffee industry is facing a sustainability problem. The chemical composition of the SCG is interesting where the organic matter represents ~90%. Cellulose, hemicellulose, galactose, and mannose are also important constituents. This huge amount of the organic matter is hazardous. Several attempts were therefore prompted to reuse the SCG, however, the power consumption, high content of lignin and the production of particulate matter represent major limitations [19].

Reusing this suppositional byproduct via a more profitable approach is therefore needed. Biochars (BC) of natural origin with their high surface area, pore volume, unique surface chemistry and liability for functionalization, are therefore a perfect fit for the physi– and chemisorption of aquatic pollutants [2,3,4,20,21,22,23]. Yet, the selectivity and adsorption capability of these biomasses are sometimes questionable. Thus, further modification of the BC via magnetization or impregnation with nanoparticles, e.g., titanium oxide (TiO_2_) serves to improve the surface properties, adsorption kinetics and hence enhance the adsorption efficiency [24,25,26,27,28]. Few investigations were reported in literature for the application of the SCG in the remediation of the pharmaceutical wastewater (Supplementary Materials Table S1 [29,30,31,32]).

In the current work, removal of BALX from artificially polluted water samples via a practical adsorption approach was the task undertaken. In this report the synthesis and characterization of the BC obtained from spent coffee grounds (SCBC) and its subsequent impregnation using TiO_2_ nanoparticles (TiO_2_@SCBC) will be approached. Both candidates will be used for the removal of BALX from wastewater and their efficiencies will be compared.

Optimization of the variables that could affect the adsorption process using either adsorbent will be performed using a multivariate approach, Plackett-Burman design (PBD) [33,34]. The target will be set to achieve the maximum percentage removal (%removal ‘%R’ and adsorption capacity ‘*q_e_*, mg/g’) with the lowest possible utilization of resources and efforts. Statistically significant variables will be decided upon using the suitable charts together with the analysis of variance (ANOVA). Moreover, adsorption isotherms and kinetics will be studied using the suitable models.

## 2. Materials and Methods

### 2.1. Materials and Reagents

Analytical grade chemicals were used throughout this study and were used as received without further purification. BALX was a product of Biosynth^®^ Carbosynth Ltd. (Compton, Berkshire, UK). Hydrochloric acid, nitric acid, sulfuric acid, acetic acid, sodium hydroxide, sodium chloride, sodium carbonate, ammonia solution (26%), ethanol, *n*-propanol, oleylamine, ammonium acetate, and titanium tetrachloride (TiCl_4_) were supplied by Sigma–Aldrich (St. Louis, MO, USA). Deionized (DI, 18.25 MΩ·cm) water used in all experiments was obtained from a Millipore-Q water system (Merck, Burlington, MA, USA). Spent coffee grounds (SCG) were obtained by brewing ground coffee (obtained locally) with hot water several times. Stock solution (100 ppm) of BALX and further dilutions were prepared in DI water and were used for the batch adsorption studies.

### 2.2. Equipment and Software

Drying of the SCG was done using an oven (Memmert, GmbH + Co. KG, Schwabach, Germany). Biochar of SCG was obtained by burning in a Thermolyne ^TM^ furnace (Barnstead, Dubuque, IA, USA) at 500 °C. A pH meter (Vernier Labquest, Beaverton, OR, USA) was used to measure the pH of water in which the adsorbents were suspended. pH values were adjusted using either 0.1 M HCl or 0.1 M NaOH. A centrifuge (Thermo Fisher Scientific, Waltham, MA, USA) was utilized to help separating the adsorbent from the adsorbate. Millex syringe filters were used (nylon, non-sterile, 0.45 µm) to filter the BALX–adsorbents suspensions. To measure the absorbance and hence the concentrations of BALX in the filtrates, a UV-Vis spectrophotometer (Agilent diode-array, Agilent, Santa Clara, CA, USA) with 10 mm matched quartz cuvettes was utilized.

Surface functionalities of both adsorbents were determined using FTIR spectroscopy (FTIR, Perkin Elmer, Shelton, CT, USA). Morphologies of both adsorbents was examined using a scanning electron microscopy (SEM, FEI, Quanta 200, Thermo Fisher Scientific) equipped with energy-dispersive X-ray spectrometer (EDS). The later was operated to study the elemental composition of both adsorbents. Raman spectroscopic analysis (Thermo Fisher Scientific) was used to investigate the nature of the produced biochars. Transmission electron microscope (TEM, TECNAI G2 TEM, TF20 FEI, Hillsboro, OR, USA) was employed for microstructural characterization of both adsorbents. Surface features such as pore size, surface area and volume were determined operating an ASAP2020 accelerated surface area and porosimetry system (Micrometrics, Norcross, GA, USA). Samples were first degassed then the N_2_ adsorption–desorption was studied. To find the surface area and the pore volume, the isotherms measured at 77 K were utilized together with the Brunauer–Emmett–Teller (BET) equation and using the t-plots with Barrett–Joyner–Halenda (BJH) equations. Minitab^®^19 software (Minitab Inc., State College, PA, USA) was used to generate the PBD and analyze the obtained data following the batch adsorption experiments.

### 2.3. Preparation of Spent Coffee Grounds Biochar (SCBC)

The coffee grounds were boiled five times with hot water and then twice with DI water. Obtained SC grounds were dried at 80 °C for three days. To obtain the BC, grounds were then burnt at 500 °C for 1 h in the oven. The burnt coffee sample was then divided into two portions after sieving using a 0.125 mm sieve. The first portion was labelled as the spent coffee biochar (SCBC) and was kept in sealed bottles for further use.

### 2.4. Preparation of the Titanium–Impregnated Spent Coffee Biochar (TiO_2_@SCBC)

The TiO_2_ nanoparticles were prepared following the hydrothermal method with minor modifications [26,35,36], where the 0.1 M oleylamine solution was used to help controlling the particle size of the formed nanoparticles [37]. The TiO_2_@SCBC was prepared by mixing 5 g of the SCBC (second portion) with 200 mL of DI water, followed by adding 100 mL of 0.1 M oleylamine solution dissolved in *n*-propanol, and the solution was then heated to 70 °C. An aliquot, 17 mL of TiCl_4_ (0.09 M) was then added to the mixture, and the pH of the mixture was adjusted to pH ~1.8 using ammonia solution (26%). The solution was stirred for 2 h at a constant temperature (70 °C) and a fixed stirring speed (700 rpm). Next, the pH of the reaction mixture was adjusted to pH ~8 using ammonia solution; and the mixture was left to cool down to the room temperature with continuous stirring for 20 h. Finally, the resultant product was centrifuged at 4000 rpm, washed three times with DI water, dried in an oven at 80 °C overnight, and the final product was labelled as TiO_2_@SCBC.

### 2.5. The Point-of-Zero-Charge (PZC)

To find the PZC, a series of 0.01 M NaCl solutions were prepared at initial pH values (pH_i_) of 3.0–9.0 ± 0.2 using either 0.1 M HCl or 0.1 M NaOH. Aliquots of 25 mL of these solutions were mixed with 0.2 g of the SCBC. Samples were left for 48 h in the automatic shaker at 150 rpm. The final pH of these solutions was then measured (pH_f_). The pH_f_ was then plotted versus the pH_i_, and the intersection of the horizontal section of this plot with the pHi axis is the pH_PZC_ value [38].

### 2.6. Batch Adsorption Studies

In the current study, a two-level fractional factorial screening design, PBD, was employed to assess the significance of four variables on the adsorption efficiency. The assessed variables are listed in Table 1 and the experimental scenario following PBD is exhibited in Table 2.

As per the shown scenario, twenty–eight experimental runs including eight central points (Ct Pt, where all variables are assessed at their mid–levels) were performed over three blocks. Two responses were measured, and the objective was to maximize these responses individually. Values of the measured responses were calculated using Equations (1) and (2) [33,34]:

(1) $$\left(\%R\right)=\frac{C_{in}-C_{eq}}{C_{in}}\;\times \;100\%$$

(2) $$\left(q_{e}\right)=\frac{C_{in}-C_{eq}}{W}\;V$$

where *C_in_* (ppm) signifies the initial drug concentration, [BALX], *C_eq_* (ppm) is the concentration of the [BALX] at equilibrium, V symbolizes the volume of the solution (L), and W represents the weight of the SCBC or TiO_2_@SCBC used (g).

### 2.7. Sorption Equilibrium and Kinetics

To study the sorption equilibrium, a drug stock solution of 500 ppm was prepared. Serial dilutions from the stock in the range of 5–400 ppm were then prepared in DI water, and the pH was adjusted to pH 9.0 ± 0.2 using aqueous solution of 0.1 M NaOH. An amount of the adsorbent (SCBC or TiO_2_@SCBC) of 0.100 ± 0.005 g was added to 13 mL of the previously prepared solution. The adsorbent suspensions were then disposed in the automatic shaker and samples were left to equilibrate for 24 h at 150 rpm. Samples were filtered, and the absorbance was measured at 290 nm. To study the kinetics of the adsorption process, aliquots of 200 mL of BALX solution (200 ppm, pH 9.0 ± 0.2) were mixed with ~1 g of either adsorbent with shaking. An aliquot of 10 mL was taken out over a total duration of 2 h. The solution was filtered, and the absorbance of the obtained filtrate was measured at 290 nm.

### 2.8. Desorption and Regeneration Experiments

To investigate the probability of the reusability of the tested adsorbents, an amount of 2.0 g of SCBC was initially equilibrized with a 220 mL of 30 ppm BALX solution for a period of 2 h at room temperature. The adsorbent–adsorbate suspension was then filtered. The adsorbent was rinsed with DI water to eliminate any free BALX, dried in the oven at 70 °C for 48 h. Six eluents were tested in this investigation, mainly 0.1 M aqueous solutions of HCl, H_2_SO_4_, HNO_3_, Na_2_CO_3_, as well as ethanol and DI water. Desorption studies were done by mixing an amount of 0.1 g of the BALX—laden adsorbent with 10 mL of each eluent. Prepared samples were then set aside in the shaker for 30 min at 150 rpm. Samples were filtered, and the absorbance of the filtrate was measured at 290 nm. All desorption experiments were repeated three times each and the average of the desorbed amount was calculated. Error bars were utilized to show the standard deviation between the replicate measurements. Following the desorption study findings, 0.1 M HNO_3_ was further used to conduct the recovery investigation. An amount of 0.2 g of the tested adsorbent was first equilibrized with 25 mL of 30 ppm BALX solution (pH 9.0 ± 0.2) for 1 h at room temperature. The resultant suspension was filtered, and the absorbance of the filtrate was measured at 290 nm. A solution of 0.1 M HNO_3_ was used to elute the loaded adsorbent and samples were then left in the oven at 70 °C for 1 h, then used for the subsequent adsorption cycle. This process was repeated for five cycles, and in each cycle, the removal efficiency (%R) was calculated.

### 2.9. Economics and Financial Assessment

To evaluate the financial efficiency of the approach used for the biochar production via recycling of food wastes, it is essential to take into account the expenses of all materials along with the energy expenditures. In contrast to the commercially available sorbents, food wastes (spent coffee grounds in this investigation) are insignificant, so it will not be considered as part of the financial assessment.

Moreover, upcycling of a waste material into a profitable product helps to alleviate the environmental problem that could be encountered when these waste materials are not properly used. The estimated energy consumption per kg of the biochar (SCBC) is ~174.6 KWh/kg for a power cost of ~0.088 $/KWh. This encompassed the energy expenditure by both the oven and the furnace, used for drying and burning the SCG, respectively. The overall cost/kg is therefore ~15.36 $ compared to an ordinary price of 124 $/0.5 kg (Sigma–Aldrich website, and this cost does not include the shipping fees to Qatar) [39].

## 3. Results and Discussion

### 3.1. Physicochemical Characterization

#### 3.1.1. Thermogravimetric Analysis (TGA)

**Figure 1:**
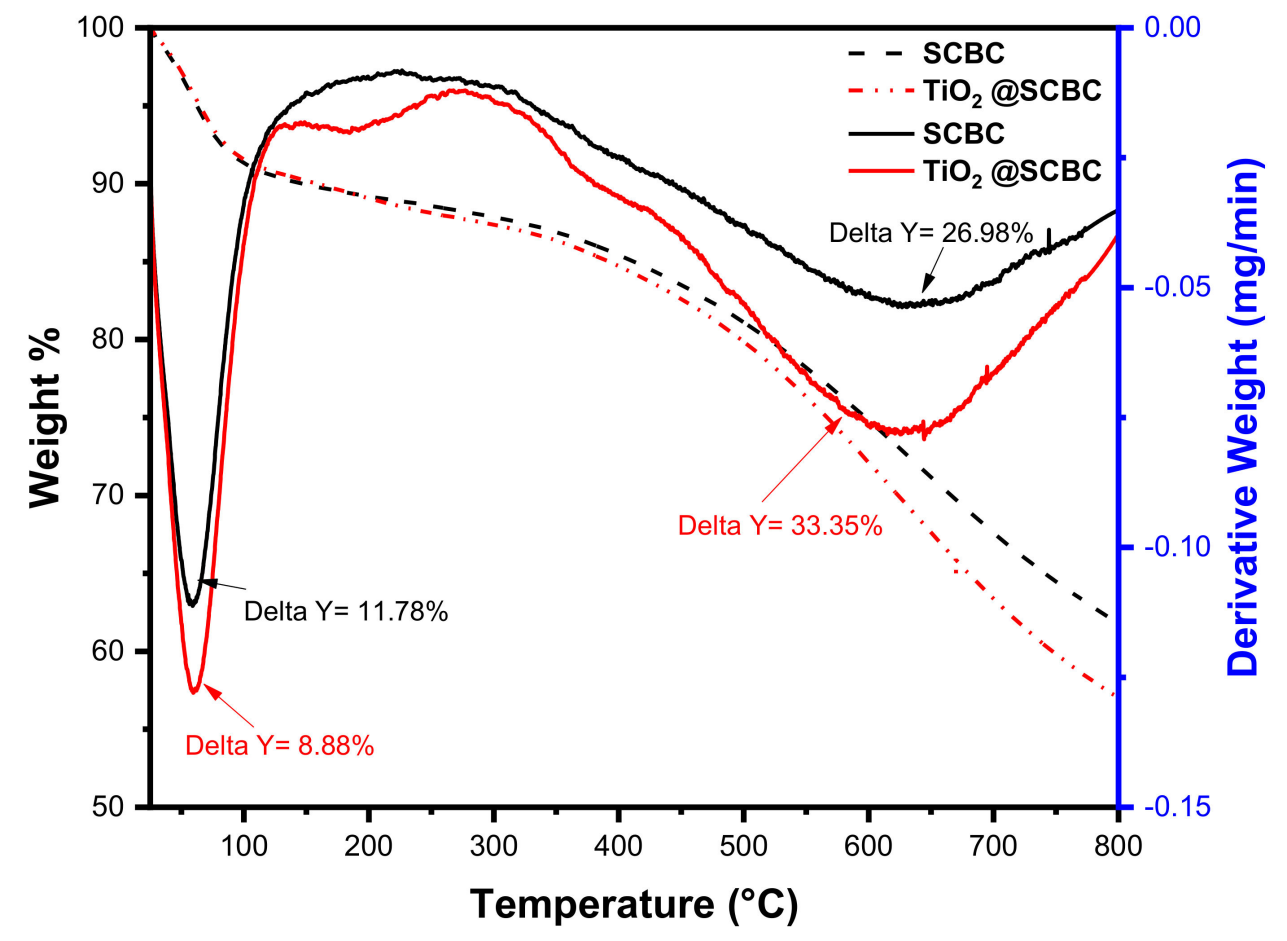
TG/*d*T spectra of SCBC and TiO_2_@SCBC.

The thermogravimetric spectra (TG) as well as the differential thermal (*d*T) spectra of both adsorbents in the range of 25–800 °C are shown in Figure 1. Obtained data show that the weight loss below 100 °C was 11.78% and 8.88% for SCBC and TiO_2_@SCBC, respectively, resulting from the vaporization of free (physically attached) water. Weight loss at a temperature higher than 100 °C and less than 300 °C was more obvious in case of TiO_2_@SCBC compared to the SCBC. This loss could be attributed to the chemical change and the conversion of some titanium hydroxide into the oxide via loss of water from the hydroxyl groups. The weight loss between 350–800 °C was higher in case of TiO_2_@SCBC (33.35%) compared to the SCBC (26.98%). This weight loss is probably due to the loss of the organic matter and the subsequent carbonization of the polymeric material in both adsorbents. The increased loss in case of TiO_2_@SCBC could be attributed to the phase transition of the adsorbent material [40].

#### 3.1.2. Fourier–Transform Infrared (FTIR) Spectra

**Figure 2:**
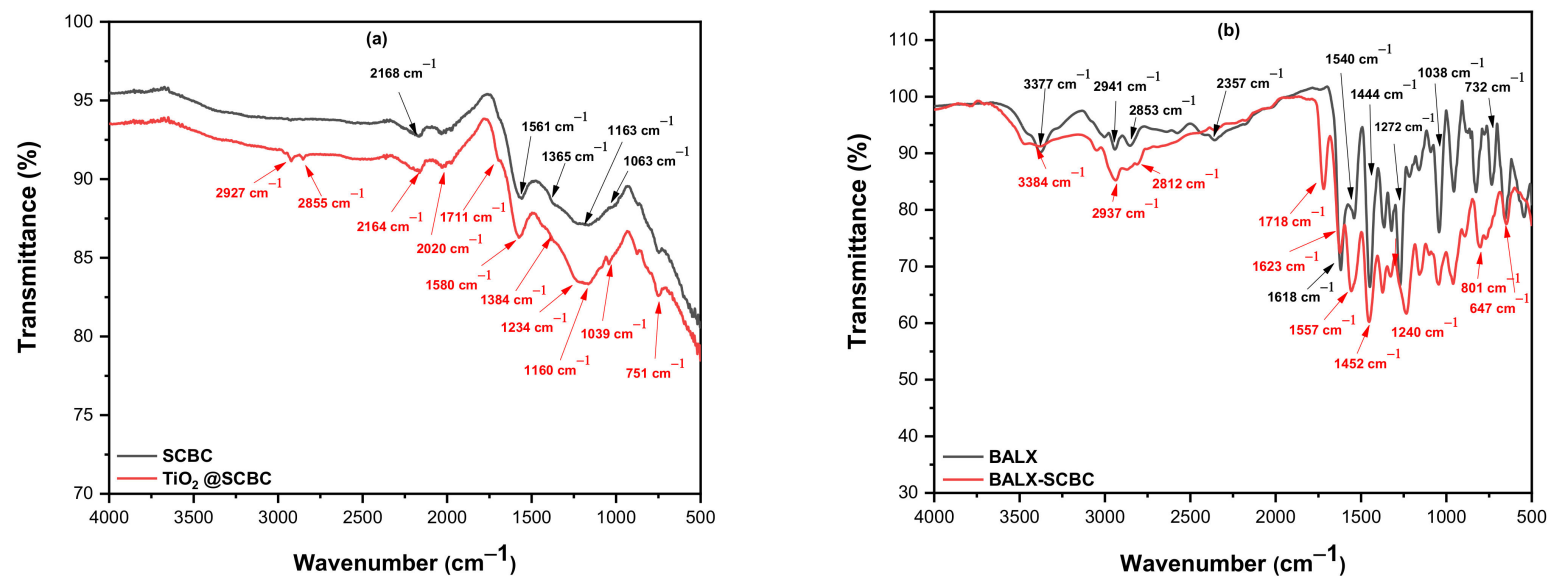

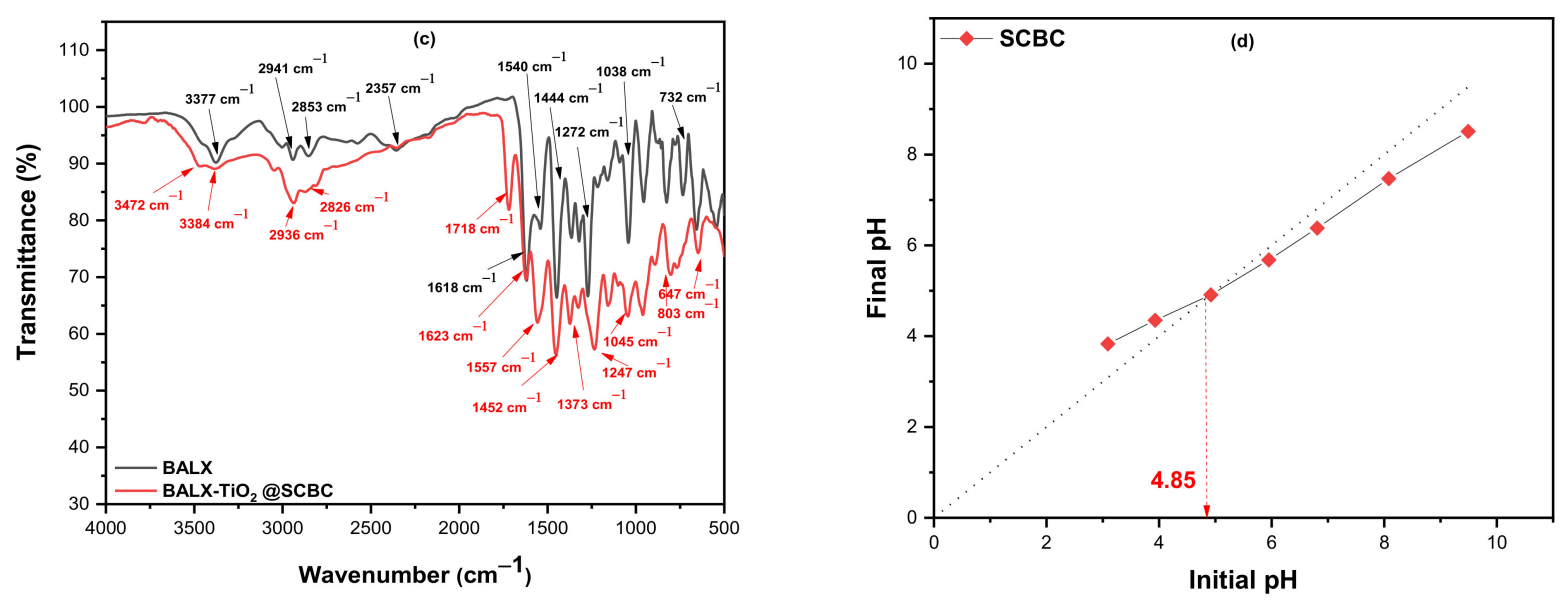
(**a**) FTIR spectra of the two adsorbents before adsorption, (**b**) BALX and SCBC after adsorption, (**c**) BALX and TiO_2_@SCBC after adsorption and (**d**) the pH_pzc_ of the SCBC sample.

FTIR analysis was used to investigate the functionalities on the surface of both adsorbents before and after adsorption of the target contaminant, BALX. The data presented in Figure 2a showed the existence of similar functionalities in both adsorbents with minor shifts in the band position. Spectra of both adsorbents revealed the absence of the peak at 3400 cm^−1^ which is associated with the hydrogen-bonded OH from water, implying the complete dehydration during the carbonization process. Typical moieties present in the SCG were also identified, e.g., the aliphatic C-H stretching vibration that was observed at 2927 cm^−1^ and 2855 cm^−1^, the C-C stretching vibration in alkyne that was detected at 2164 cm^−1^ and 2168 cm^−1^, the carboxyl C=O and the aromatic C=C stretching vibrations in the range of 1500–1700 cm^−1^, the N–H or the –OH stretching vibrations observed at 1365 cm^−1^ and 1384 cm^−1^ for the SCBC and the TiO_2_@SCBC, respectively. The peak at 1063 cm^−1^ is associated with the C–OH vibration [41,42,43]. The same peak appears in the spectrum of the TiO_2_@SCBC but with a red shift to 1039 cm^−1^. This shift could have resulted from the formation of a bond between TiO_2_ and the surface of the SCBC. The presence of TiO_2_ was also proved by the FTIR analysis where the two characteristic absorption peaks at 1630 cm^−1^ which could be attributed to the bending vibration modes of the Ti–OH and at 1383 cm^−1^ which is associated with the Ti-O stretching modes could be observed [44,45].

On the other hand, the FTIR spectrum of free BALX (Figure 2b,c), shows the presence of three major peaks, including two bands at 2853 and 1623 cm^−1^, which are attributed to the stretching vibration of -CH_3_ and -C=O of BALX quinolone, respectively, and a characteristic ether band at 1272 cm^−1^ [46]. Following adsorption, the obtained spectra confirm the presence of BALX onto both adsorbents, where the majority of the BALX peaks appear with minor shifts and bit different intensities. For example, the band of BALX at 2853 cm^−1^ has shifted to 2812 and 2826 cm^−1^ for the adsorbed drug on the SCBC and TiO_2_@SCBC, respectively. These findings confirm the adsorption of the BALX onto both adsorbents. 

Investigation of the charge on the adsorbent surface showed that the pH_PZC_ in case of the SCBC was 4.85 which is similar to the previously reported figures [47,48]. This value implies the existence of more acidic moieties on the surface of the SCBC compared to the basic ones. In the same itinerary, the pKa value of BALX is 6.0 ± 0.2 [10]. Therefore, and depending on the findings of the PBD for the optimum pH value, and the equilibrium data, the type of adsorption (chemi- or physisorption) could be decided.

#### 3.1.3. Raman Spectroscopic Analysis

**Figure 3:**
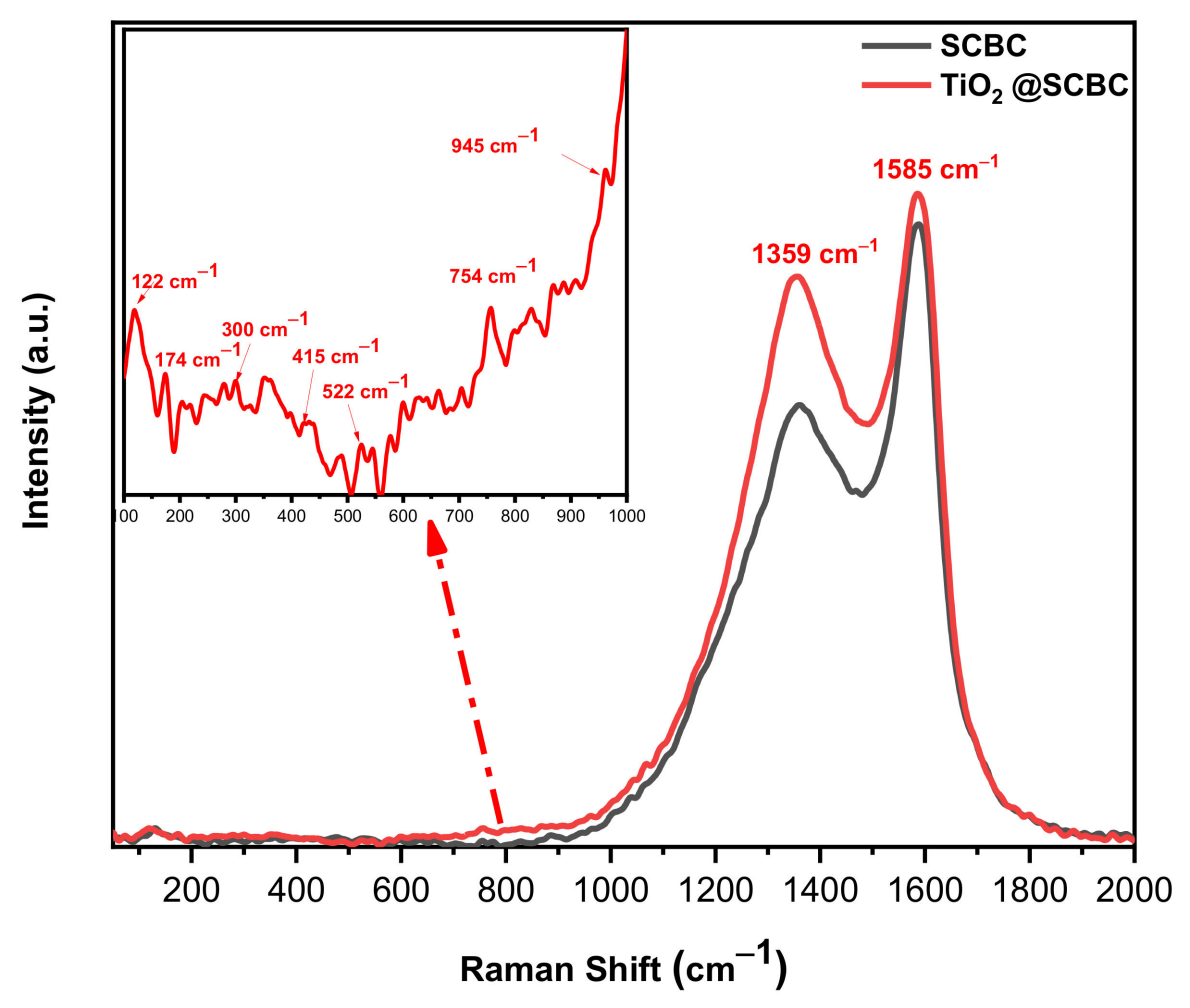
Raman spectra of the as-prepared adsorbents: SCBC and TiO_2_@SCBC.

Raman spectra of both adsorbents are shown in Figure 3. The obtained data show two prominent bands at 1359 and 1585 cm^−1^, which are associated with the D- and the G-bands, respectively, and are distinctive for the carbonaceous materials. The D–band at 1359 cm^−1^ is related to the defects of the graphitic structure (*sp*^2^ connection) [49]. However, the G-band is attributed to the C=C stretching of this graphitic structure, and it helps to determine the sample graphitization. On the other hand, the I_D_/I_G_ ratio can be used to determine the degree of the defect on the surface of both adsorbents. The obtained data show that the I_D_/I_G_ ratio for SCBC was 0.71, while for the TiO_2_@SCBC, it was 0.87 confirming the presence of higher defects on the surface of the impregnated sample. This could be attributed to the presence of the TiO_2_ nanoparticles on the surface of the biochar. The Raman spectrum of the TiO_2_ nanoparticles was previously reported [50], where TiO_2_ spectrum was reported to have six allowed models that appear at 144 cm^−1^ (E_g_), 197 cm^−1^ (E_g_), 399 cm^−1^ (B_1g_), 513 cm^−1^ (A_1g_), 519 cm^−1^ (B_1g_), and 639 cm^−1^ (E_g_). Figure 3 shows weak bands in the range between 100–700 cm^−1^ in case of the TiO_2_@SCBC, which could be attributed to the TiO_2_ nanoparticles. However, this cannot be confirmed, most likely because the concentration of TiO_2_ on the surface was very low, and therefore the bands are very weak. Yet, the presence of the TiO_2_ on the surface was confirmed by the FTIR analysis and will be further verified using the SEM and TEM analyses.

#### 3.1.4. Pore Structure Characterization of the Prepared Adsorbents

**Figure 4:**
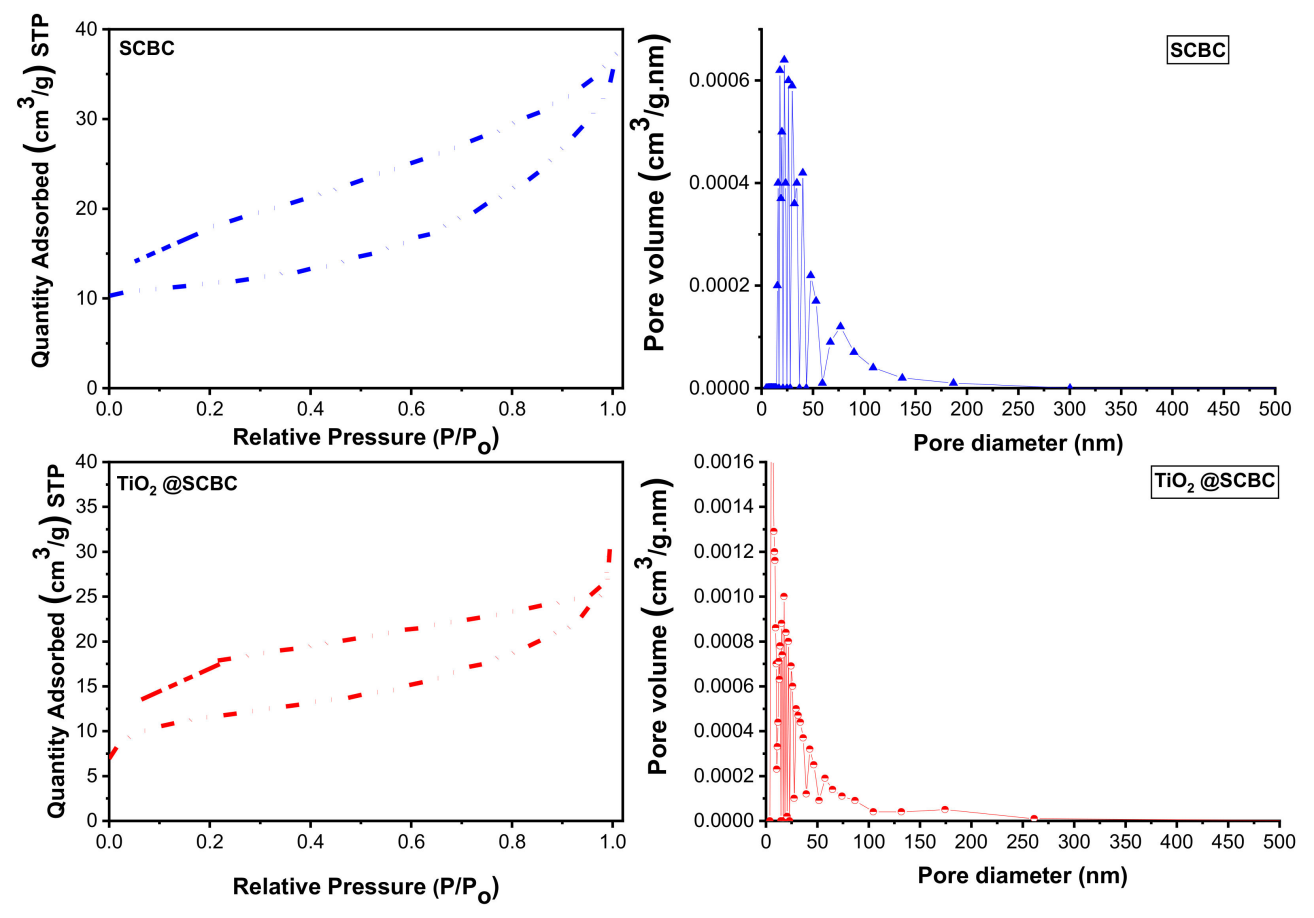
BET analysis of SCBC and TiO_2_@SCBC samples.

BET analysis output of the two adsorbents is shown in Figure 4 and Table 3. The obtained data show that the Langmuir surface area of SCBC was 49.23 m^2^/g, compared to 50.54 m^2^/g in case of the TiO_2_@SCBC. The surface area did not increase significantly following the impregnation process most likely because the concentration of TiO_2_ used was not that high. The obtained data revealed the presence of two types of pores in both adsorbents: mesopores (2–50 nm) and macropores (pore size is >50 nm). The average pore volume in case of the SCBC was a bit higher compared to the TiO_2_@SCBC, and the ratio of macropores to mesopores for the SCBC sample was higher compared to the impregnated sample. This finding could be attributed to the coverage of some pores by the TiO_2_ nanoparticles. The adsorption isotherm type was of type II for both adsorbents, implying that the micropores were filled with nitrogen gas at low pressure. Monolayer formation is starting to be formed at the knee, followed by multilayer formation at medium pressure, but at the higher pressures, capillary condensation has occurred. Furthermore, both adsorbents showed a hysteresis loop of the H4 type, which is usually found on solids with an extensive pore size distribution [51,52].

#### 3.1.5. SEM, EDS, and TEM Analyses

**Figure 5:**
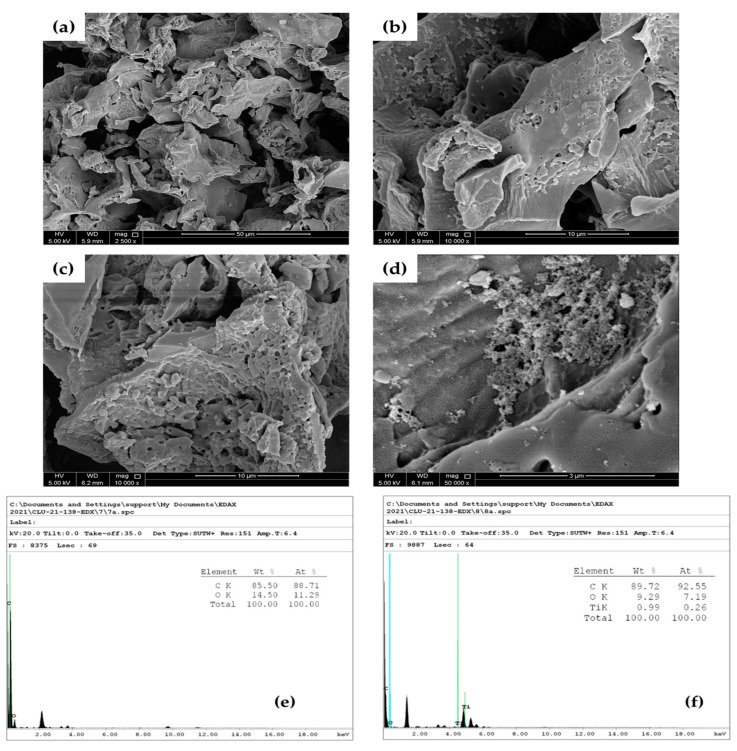
SEM micrographs of (**a**,**b**) the SCBC (at 2500× and 10,000× magnifications), (**c**,**d**) the TiO_2_@SCBC (at 10,000× and 50,000× magnifications), (**e**,**f**) EDS analyses of the SCBC and the TiO_2_@SCBC, respectively.

SEM, EDS, and TEM analyses were used in this study to determine the morphology and structure of the as-prepared adsorbents. SEM micrographs of the SCBC, Figure 5a,b, reveal different types of pores as previously illustrated by the BET analysis. On the other hand, the SEM micrographs of the TiO_2_ impregnated SCBC, Figure 5c,d, show the presence of small particles of the TiO_2_ nanoparticles on the surface of the SCBC. The SEM findings were further confirmed by the EDS analysis, Figure 5e,f, where the titanium concentration was found to be 0.99% in case of the TiO_2_@SCBC (absent in case of the SCBC sample), implying the presence of the TiO_2_ on the surface of the impregnated SCBC.

**Figure 6:**
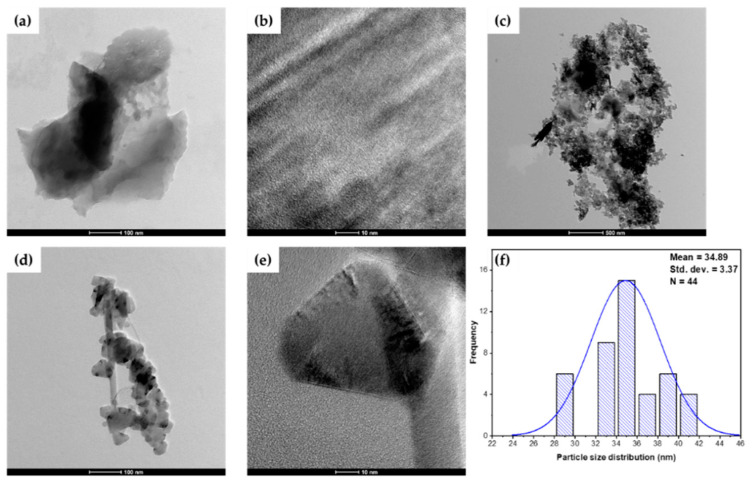
TEM images of the SCBC at two scales (**a**) 100 nm, (**b**) 10 nm, TiO_2_@SCBC at (**c**) 500 nm, (**d**) 100 nm and (**e**) 10 nm, and (**f**) PSD for the TiO_2_@SCBC.

The microstructure of both adsorbents was also studied using the TEM analysis. Figure 6a,b show that the surface of the SCBC is plain with no particles on the surface. However, the micrographs of TiO_2_@SCBC presented in Figure 6c,d, and e show pyramidal shaped TiO_2_ nanoparticles on the surface. The particle size distribution (PSD) is shown in Figure 6f and indicates that the average particle size (PS) of the as-prepared TiO_2_ nanoparticles was 34.89 ± 3.37 nm. The obtained data further confirm the findings of BET, SEM, and EDS analyses [53,54].

### 3.2. Plackett–Burman Design (PBD)

PBD, a well-known two-level factorial design, has been used to screen out the statistically insignificant variables. PBD is a cost-effective screening design, where only the main effects are considered, assuming that the other factorial interactions are insignificant if compared to the main effects [33,34,55]. Design setup is shown in Table 2.

#### 3.2.1. Screening of the Variables’ Significance

Typically, for a PBD, a first-order polynomial equation is fitted to the experimental data to estimate the effects of studied variables. Equations (3)–(6) are the obtained regression equations. The obtained models show a clear depiction of the relationship between the magnitude and the direction of impact for each independent variable and the measured dependent response:

ln(%R)_SCBC_ = 2.7144 + 0.01887 pH − 0.001594 [BALX] + 0.004205 CT + 0.008147 AD − 0.2288 Ct Pt (3)

ln(*q_e_*)_SCBC_ = −0.3570 + 0.01437 pH + 0.035295 [BALX] + 0.004218 CT − 0.007241 AD − 0.0716 Ct Pt (4)

ln(%R)_TiO__2@SCBC_ = 2.6845 + 0.07509 pH − 0.010758 [BALX] + 0.002126 CT + 0.008961 AD − 0.8109 Ct Pt (5)

ln(*q_e_*)_TiO2@SCBC_ = −0.4524 + 0.08983 pH + 0.026157 [BALX] + 0.001941 CT − 0.006676 AD − 0.6636 Ct Pt (6)

**Figure 7:**
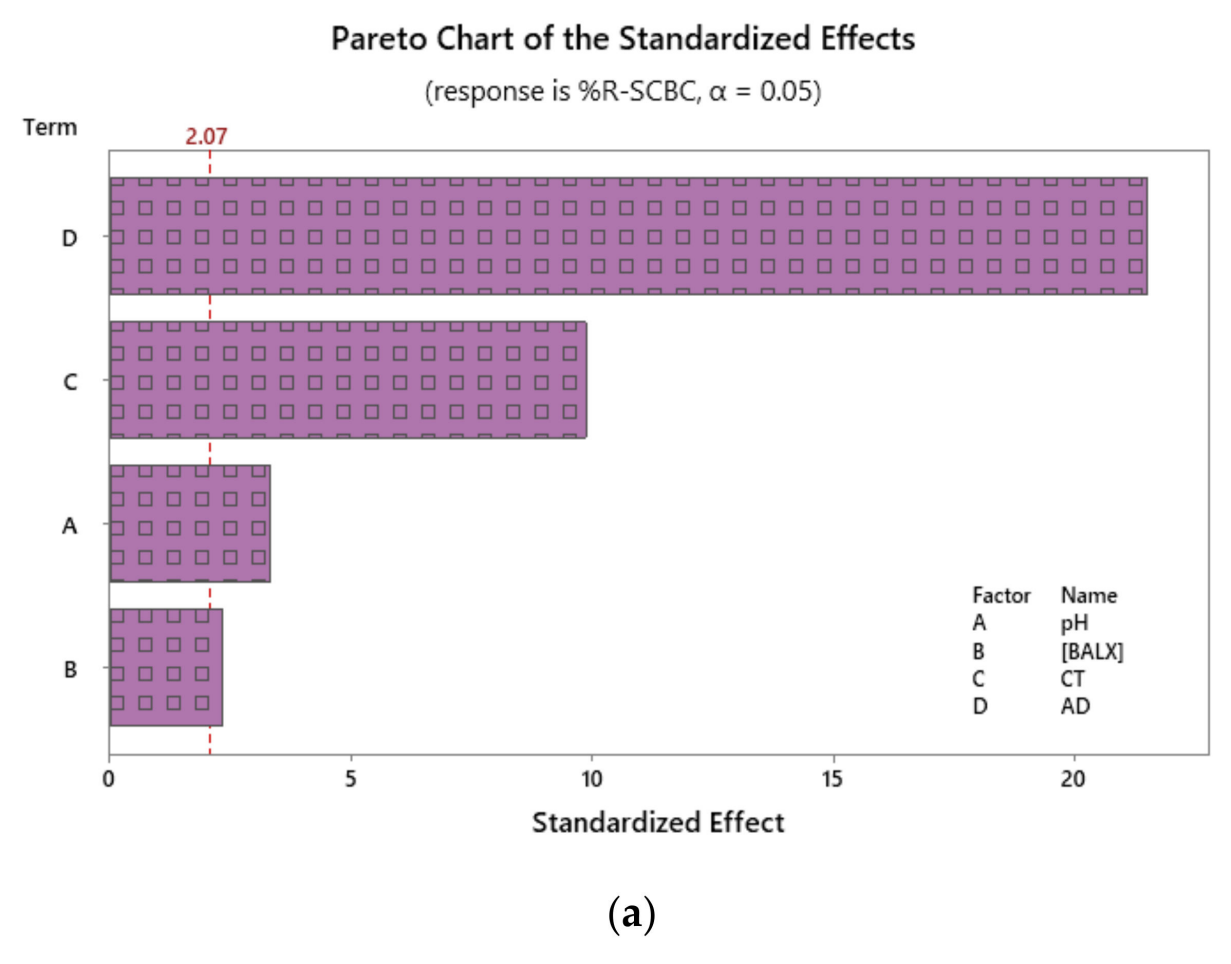

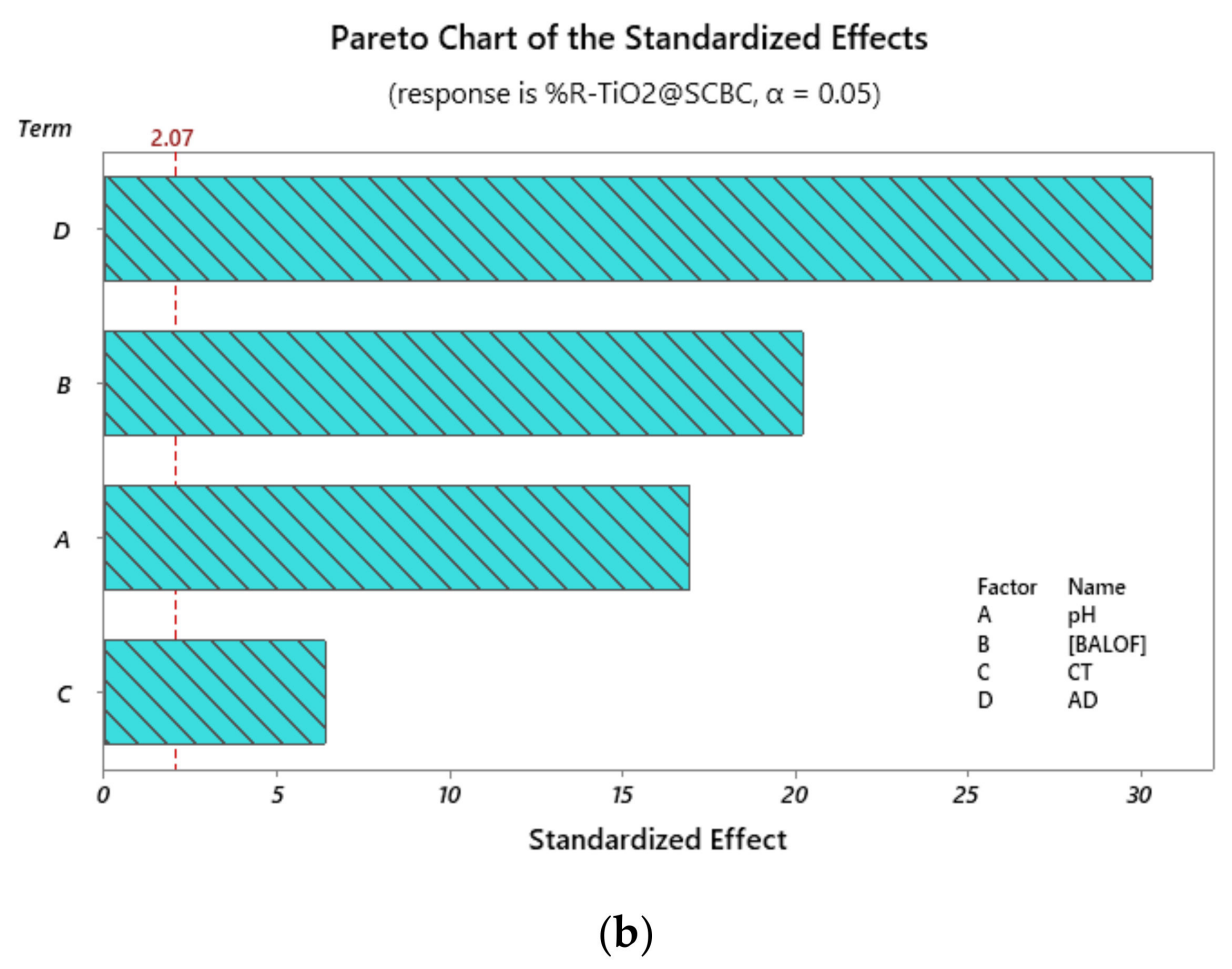
Pareto chart of standardized effects in case of %R (SCBC (**a**) and TiO_2_@SCBC (**b**)) is the response to be optimized.

A Pareto chart of standardized effects was also used to illustrate the variables’ significance (Figure 7). In case the %R_SCBC_ is the measured response, the AD (D) followed by the CT (C) were the most significant variables. Similar influence of the AD (D) was observed in case of %R_TiO2@SCBC_. In case of *q_e_*, however, the impact of [BALX] (B) followed by the AD (D) were the variables with the highest influence on the measured response. The influence of the pH (A) was of a lower impact on both adsorbents. These outcomes further substantiate the findings of the FTIR analysis and the measured value of the PZC, where pH has nearly no influence on the adsorption efficiency. This finding further supports the possibility of occurrence of physisorption rather than chemisorption specially in case of the SCBC.

In the same itinerary, and to assess the statistical significance of the tested variables, analysis of variance (ANOVA) testing was instigated (Table 4). Via ANOVA testing, the *p*-value for each term was compared to the significance level (95.0 confidence level ‘CI’, *p* = 0.05) to evaluate the null hypothesis. As shown in Table 4, terms with *p*-value ˂ 0.05 are statistically significant.

#### 3.2.2. Model Fitting Parameters

To determine how well the obtained regression models, Equations (3)–(6), fit the obtained data, four parameters are usually considered; (1) Root mean square error (RMSE), (2) Coefficient of determination (R^2^), (3) R^2^−adjusted (R^2^−adj), and (4) R^2^–predicted (R^2^–pred). Table 5 shows the last three parameters for the four generated models. As shown, the values of R^2^ and R^2^−adj were high enough implying the goodness-of-fit. The value of R^2^–pred was utilized to determine how well the model foresees the response for new observations. As also shown, the R^2^–pred was not substantially less than the value of the R^2^, confirming that the models were not over-fit. It is noteworthy to mention that the difference between the predicted and the observed responses was assessed using the RE, Table 2. Obtained values of RE were small implying that developed models are accurate.

#### 3.2.3. Contour, Surface, and Optimization Phase Plots

**Figure 8:**
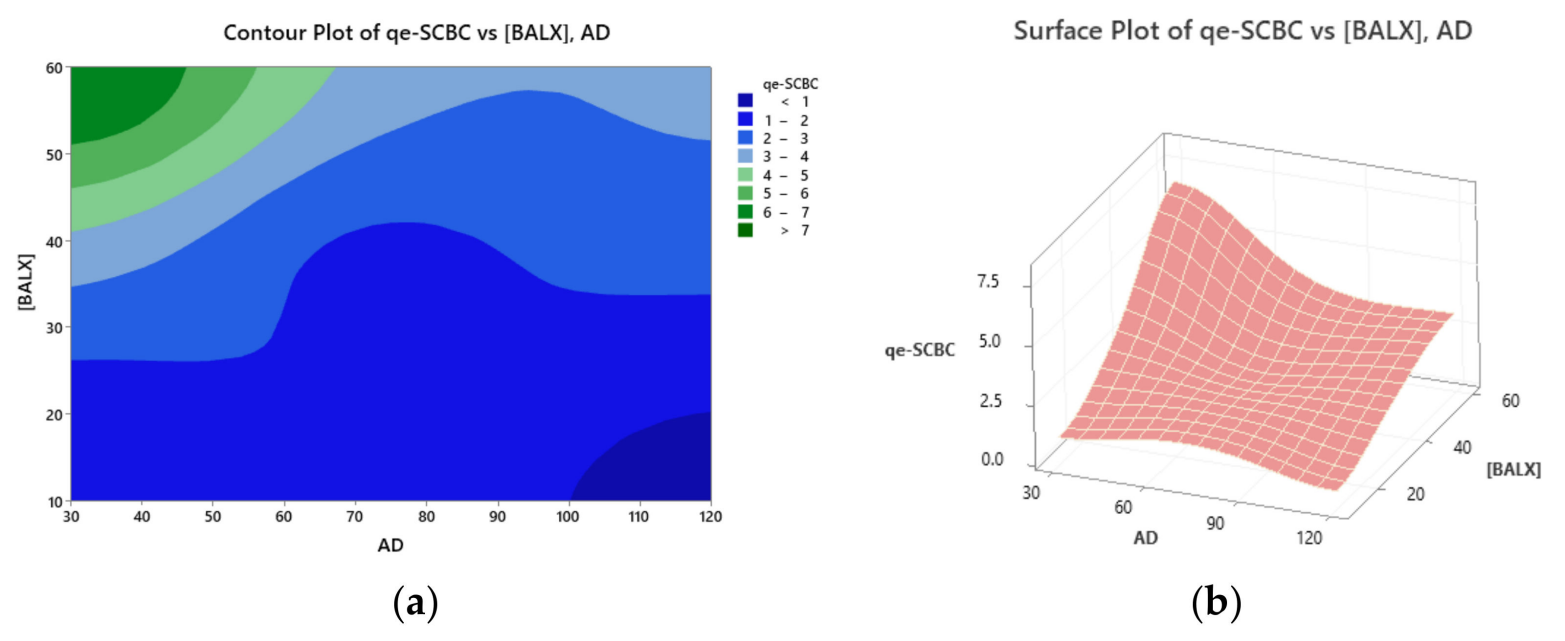
Sample contour (**a**) and surface plots (**b**) in case the *q_e_* (mg/g) is the response and using SCBC as adsorbent.

The 2D contour plots were used to plot the liaison between a fitted response (dependent variable) and two continuous independent variables. A sample contour plot is shown in Figure 8–left panel. The figure displays a two-dimensional view in which points with the same *q_e_* (mg/g) value are connected to produce the contour lines. As per the legend, a value of *q_e_* = 6–7 mg/g could be obtained when the [BALX] is between 52–60 ppm and the AD is 30–45 mg.

The 3D surface plots, similarly, relates a fitted response to two continuous variables, however, the relationship is in a three-dimensional format were used to ascertain the impact of certain set of variables on the measured response (s). Sample surface plot is shown in Figure 8—right panel. As shown, a value of *q_e_* > 7 mg/g could be achieved using a [BALX] > 50 ppm and an AD~30 mg.

Following the screening process using the PBD, optimization was made using the response optimizer tool. The factorial blends that could boost the measured response are displayed in Table 5. Assessment of the blends shown in Table 5 is done considering the value of the desirability function (*d*), where the nearer the value of *d* to 1.000, the better the blend [56].

### 3.3. Isotherms and Adsorption Kinetics

The adsorption potential of an adsorbent is usually reliant on the existence of functional groups on the surface of the adsorbent and the surface area of the adsorbent as well as its pore structure. Considering the characterization data alongside with the PBD findings, the interaction of BALX and both adsorbents could be interpreted.

#### 3.3.1. Isotherms

The adsorption equilibrium studies are helpful in determining the type of interaction between the target pollutant and the adsorbent. Likewise, findings of the equilibrium studies could be used to identify the extent of the accumulation of the adsorbate onto the adsorbent surface. In this work, four equilibrium isotherms were used to investigate the adsorption of BALX onto SCBC and TiO_2_@SCBC, mainly Langmuir, Freundlich, Temkin, and Dubinin-Radushkevich (D-R) isotherms [57,58,59,60,61].

The data of the Langmuir equilibrium isotherm could be related to three hypotheses:

**Hypotheses** **1.** *The adsorption energy of the adsorption sites on the studied adsorbent is constant*.

**Hypotheses** **2.** *No interaction between the adsorbate molecules and each adsorbate molecule could occupy only one site on the surface of the adsorbent*.

**Hypotheses** **3.** *The adsorption process is localized*.

**Figure 9:**
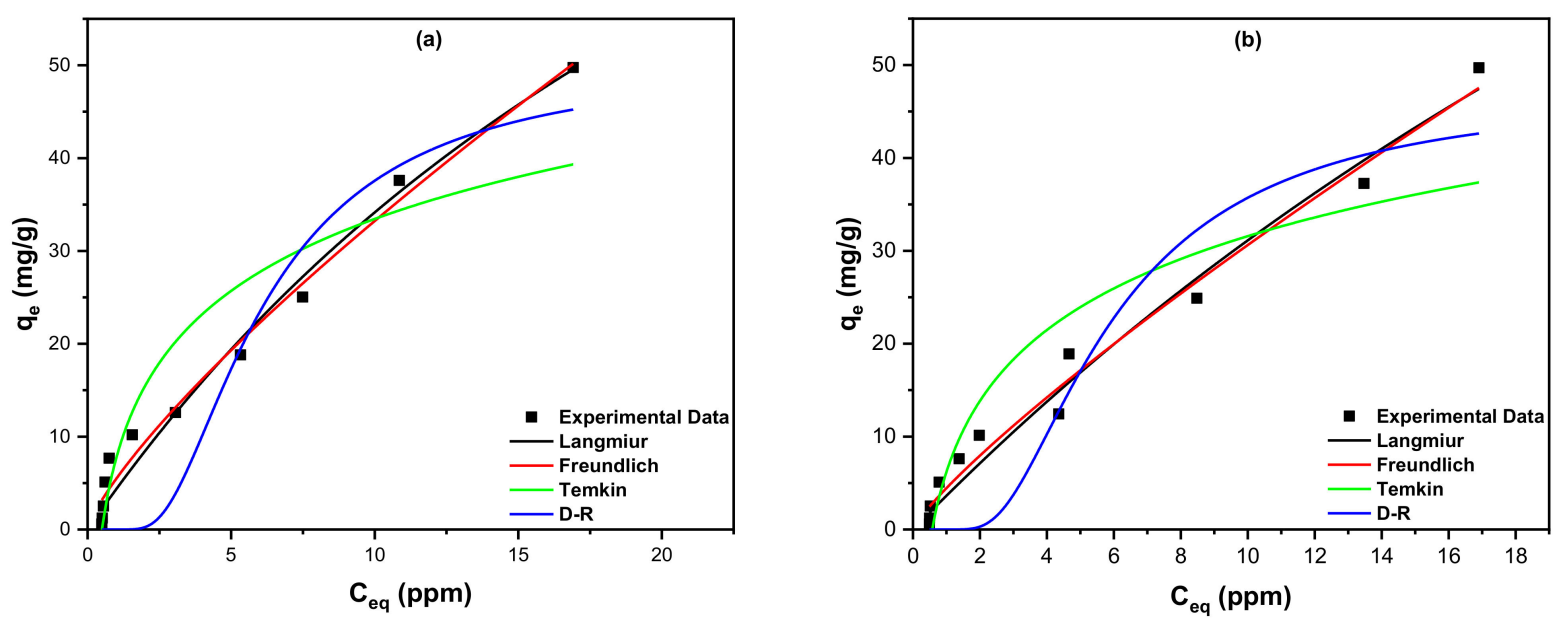
Adsorption isotherms of BALX onto (**a**) SCBC and (**b**) TiO_2_@SCBC including Langmuir, Freundlich, Temkin, and Dubinin-Radushkevich (D-R) isotherms.

The Langmuir isotherm for the adsorption of BALX onto both SCBC (Figure 9a) and TiO_2_@SCBC (Figure 9b) can be presented using Equation (7) as follows:

(7) $$q_{e}=\frac{q_{m}\;K_{L}\;C_{eq}}{1+K_{L}\;C_{eq}}$$

where *q_m_* is the maximum adsorption capacity and *K_L_* is the Langmuir equilibrium coefficient. The Langmuir equation can be presented using the following dimensionless formula:

(8) $$R_{L}=\frac{1}{1+K_{L}\;C_{in}}$$

where *R_L_* is the separation factor, and *C_in_* (ppm) is the BALX initial concentration. The *R_L_* value was used to find the adsorption favorability of adsorbate onto adsorbent; thus, if *R_L_* is >1, then the adsorption process is unfavorable, while if the *R_L_* = 1, then the adsorption is linear and if the value is between 0–1, the adsorption is favorable and spontaneous. On the other hand, if the *R_L_* = zero, then the adsorption process is irreversible. The calculated *R_L_* value for SCBC and TiO_2_@SCBC was ˂1, signifying that the adsorption process of BALX onto both adsorbents was spontaneous. The maximum adsorption (*q_max_*) for both adsorbents was found to be 142.55 mg/g for SCBC and 196.73 mg/g for TiO_2_@SCBC, showing that the presence of TiO_2_ nanoparticles has increased the adsorption efficiency of the SCBC towards BALX.

The Freundlich isotherm can be used to describe the energy of any heterogeneous surface, and it is presented by Equation (9):

(9) $$q_{e}=K_{F}C_{eq}^{\frac{1}{n}}$$

where *C_eq_* is the BALX equilibrium concentration (ppm); *q_e_* is the amount of BALX adsorbed/unit mass (mg/g), *K_F_* (mole/g) (L.mole^−1^)^1/*n*^ and 1/*n* are the Freundlich coefficients, Figure 9a,b for SCBC and TiO_2_@SCBC, respectively and Table 6. The obtained data showed a good fit with an R^2^ = 0.9844 and 0.9831 for both SCBC and TiO_2_@SCBC, respectively, which is higher than the other equilibrium isotherms. Hence, Freundlich isotherm can be used to explain the adsorption of BALX onto the two adsorbents. Additionally, the obtained data for SCBC show that *n* = 1.28, while for the TiO_2_@SCBC, *n* = 1.19. Consequently, the adsorption potential for SCBC and TiO_2_@SCBC (A = *n*RT) was 4.22 and 3.92 kJ. Therefore, any BALX molecule that has a potential energy lower than these values can be adsorbed on the surface and the adsorption reaction tends to be favorable and irreversible.

The Temkin equilibrium isotherm (Figure 9, and Table 6), helps to draw an idea about the BALX-SCBC/TiO_2_@SCBC interactions. Thus, the heat of adsorption of the adsorbed molecule in a layer could decrease linearly with the SCBC/TiO_2_@SCBC-BALX interactions. According to the obtained data, the sorption energy equals 221.6 and 224.7 J/mol for the SCBC and TiO_2_@SCBC, respectively, implying a favorable adsorption of BALX onto the two adsorbents and confirming the obtained data from the Langmuir isotherms.

The Dubinin-Radushkevich (D-R) equilibrium isotherm was studied at room temperature (Figure 9, and Table 6). The obtained data for both adsorbents show that the sorption energy for SCBC is 6.93 kJ/mol and for TiO_2_@SCBC is 7.08 kJ/mol. These numbers mean that the adsorption of BLAX onto SCBC is physisorption and it depends mainly on the surface area and the presence of mesopores and macropores on the surface of the biochar. On the other hand, the adsorption of BALX onto the TiO_2_@SCBC is chemisorption and the adsorption mechanism depends mainly on the presence of different functional groups on the surface of the adsorbent, as shown in the FTIR analysis. However, the occurrence of physisorption cannot be ignored because of the large surface area and the presence of a different types of pores on the surface of the TiO_2_@SCBC.

#### 3.3.2. Adsorption Kinetics

**Figure 10:**
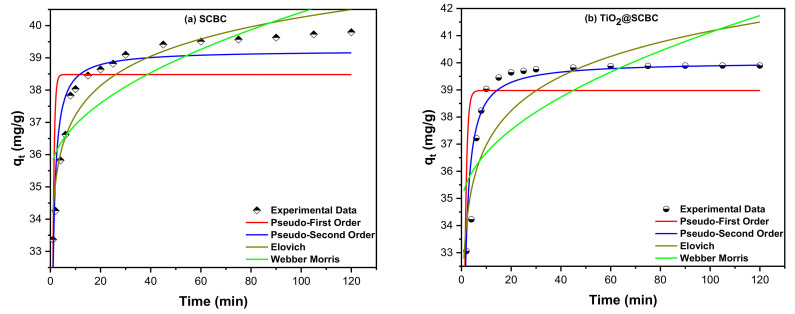
Kinetics of the adsorption of BALX onto (**a**) SCBC and (**b**) TiO_2_@SCBC including PSO, PSO, Elovich, and Weber–Morris models.

Adsorption kinetics of BALX onto the two adsorbents were investigated using four kinetic models, namely pseudo-first order (PFO), pseudo-second order (PSO), Elovich, and Weber-Morris (W-M) models. The relation between *q_t_* (mg/g) versus time (min) was plotted, Figure 10. The obtained data for the calculated parameters for these models are exhibited in Table 7. For the PFO and PSO models, the obtained data show that the R^2^ value is higher for the PSO model compared to the PFO for both adsorbents, 0.8705 for SCBC and 0.9451 for TiO_2_@SCBC. Therefore, the rate of reaction between BALX and both adsorbents is dependent mainly on both BALX and the adsorbent, and the overall reaction can be represented as follows:

(10) $$\mathrm{BALX}+{\mathrm{SCBC}\;\mathrm{or}\;\mathrm{TiO}}_{2}@\mathrm{SCBC}\;\left(\overset{k}{\to }\right)\;\left\{\mathrm{BALX}-\mathrm{SCBC}\right\}\;\mathrm{or}\;\left\{\mathrm{BALX}-{\mathrm{TiO}}_{2}@\mathrm{SCBC}\right\}$$

On the other hand, the Elovich model exhibits significant initial adsorption for both adsorbents that equals 2.44 × 10^11^ and 1.18 × 10^8^ mg g^−1^ min^−1^ for SCBC and TiO_2_@SCBC, respectively. Lastly, the R^2^ value of the W-M model was too small for both adsorbents compared to the other models (0.6863 for SCBC and 0.4910 for TiO_2_@SCBC). Therefore, this model cannot be used to represent the adsorption of BALX onto these adsorbents.

### 3.4. Desorption and Recovery Studies

**Figure 11:**
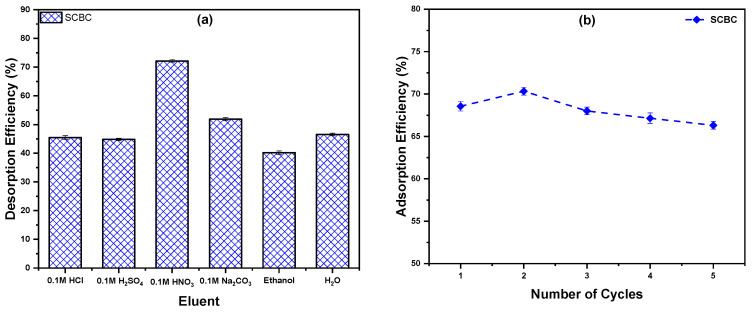
(**a**) Effect of the eluent type on the BALX desorption from the SCBC, and (**b**) the regeneration performance of SCBC for BALX removal.

The regeneration of the adsorbent is an essential aspect of its economic usability. Accordingly, a desorption study was performed using six different eluents for the BALX from a BALX-loaded SCBC sample followed by consecutive adsorption–desorption cycles repeated for five cycles. The obtained data for the desorption study are shown in Figure 11a and display the relation between the type of eluents versus the desorption efficiency (%). The obtained data show that the highest desorption efficiency for BALX from a BALX-loaded SCBC sample was accomplished using 0.1 M HNO_3_ with a desorption efficiency of 72.14%. Accordingly, 0.1 M HNO_3_ was further used as the most suitable eluent for desorbing BALX from the SCBC.

On the other hand, the SCBC regeneration study was done by performing cyclic adsorption–desorption experiments, as shown in Figure 11b. The collected data revealed that BALX removal efficiency decreased slightly, where it decreased from 68.56% (cycle 1) to 66.32% (cycle 5). The obtained data further confirm the stability of the studied adsorbent, and it can be regenerated successfully and used for more than five cycles with nearly the same BALX removal efficiency.

## 4. Conclusions

In accordance with the displayed data, competent and profitable adsorbents could be derived from spent coffee grounds (SCG). Two adsorbents were successfully synthesized and exploited in the current endeavor, the pristine spent coffee biochar (SCBC) and the TiO_2_-impregnated biochar (TiO_2_@SCBC). Both adsorbents showed a high adsorptive capability for the FQ antibiotic, balofloxacin (BALX) from artificially contaminated water samples. The removal efficacy was 68.34% using the SCBC, compared to 91.78% using the TiO_2_@SCBC. The TG/*dT* analysis showed that the TiO_2_@SCBC was less thermally stable compared to the pristine adsorbent. The impregnation of the BC with TiO_2_ did not significantly increase the surface area (49.23 and 50.54 m^2^/g in case of SCBC and TiO_2_@SCBC, respectively). In contrary, the pore volume and the pore radius have decreased with the impregnation. Moreover, the FTIR analysis before and following the adsorption of BALX showed the changes in intensities and shifts of some peaks corroborating the presence of BALX on the surface of the adsorbents. A multivariate platform was exploited to maximize the assessed responses (%R and *q*_e_, mg/g) employing Plackett–Burman design. The objective was to have a green approach for the removal of BALX using the lowest possible amount of energy and resources. Equilibrium isotherms using the nonlinear fittings indicated that the data could fit well to the Freundlich isotherm and that adsorption is favorable with a maximum adsorption capacity (*q_max_*) of 142.55 mg/g for SCBC and 196.73 mg/g for the TiO_2_@SCBC. The adsorption was physisorption using the pristine adsorbent compared to chemisorption using the TiO_2_@SCBC. Adsorption kinetics were best fit to the PSO model. The desorption and regeneration investigation showed that SCBC could be restored with the adsorption efficiency being retained up to 66.32% after five cycles. Utilization of materials and energy to produce 1 kg of the biochar was assessed to decide upon the economic profitability of the SCBC and obtained data indicated that the current approach is much more economic contrasted to commercially available adsorbents.

## Figures and Tables

**Table 1:** Variables and their lower, mid, and upper levels. Two dependent variables were measured.

Factors-Code	Unit	Low (−1)	Mid (0)	High (+1)
pH-(A)	pH unit	3.0	6.0	9.0
Initial [BALX]-(B)	Ppm	10	35	60
Contact Time (CT)-(C)	Min	10	50	90
Adsorbent Dosage (AD)-(D)	mg/13 mL	30	75	120
Dependent Variables	Percentage Removal (%R), Adsorption Capacity (*q_e_*_,_ mg/g)			

**Table 2:** Design of experiments employing PBD. Observed and predicted values for both responses and the calculated relative error (RE) are shown.

	Variables				SCBC						TiO_2_@SCBC					
Trial #	pH	[BALX]	CT	AD	%R Obs *	%R Pred **	RE ***	*q_e_* Obs *	*q_e_* Pred **	RE ***	%R Obs *	%R Pred **	RE ***	*q_e_* Obs *	*q_e_* Pred **	RE ***
01	9 (+)	60 (+)	10 (−)	120 (+)	45.31	45.08	0.01	2.94	2.90	0.01	43.40	45.22	0.04	2.82	3.14	0.10
02	9 (+)	10 (−)	90 (+)	120 (+)	62.14	68.34	0.09	0.67	0.69	0.04	92.47	91.78	0.01	1.00	1.00	0.01
03	3 (−)	10 (−)	10 (−)	30 (−)	22.11	20.94	0.06	0.95	0.87	0.09	19.77	22.03	0.10	0.85	0.90	0.06
04	3 (−)	60 (+)	10 (−)	120 (+)	39.83	40.25	0.01	2.59	2.66	0.03	29.22	28.82	0.01	1.90	1.83	0.04
05	3 (−)	60 (+)	90 (+)	30 (−)	27.76	27.07	0.03	7.19	7.14	0.01	15.26	15.25	0.00	3.96	3.90	0.02
06	3 (−)	60 (+)	90 (+)	120 (+)	57.29	56.35	0.02	3.76	3.72	0.01	32.13	34.16	0.06	2.09	2.14	0.02
07	6 (0)	35 (0)	50 (0)	75 (0)	30.36	28.91	0.05	1.84	1.75	0.05	16.09	15.27	0.05	0.97	0.94	0.04
08	3 (−)	10 (−)	90 (+)	30 (−)	28.40	29.31	0.03	1.26	1.22	0.03	24.34	26.11	0.07	1.05	1.05	0.00
09	6 (0)	35 (0)	50 (0)	75 (0)	27.06	28.91	0.06	1.64	1.75	0.06	14.74	15.27	0.03	0.89	0.94	0.05
10	9 (+)	10 (−)	10 (−)	30 (−)	21.59	23.45	0.08	0.93	0.95	0.02	32.98	34.56	0.05	1.42	1.55	0.08
11	3 (−)	10 (−)	10 (−)	120 (+)	48.39	43.59	0.11	0.47	0.45	0.03	50.44	49.35	0.02	0.55	0.50	0.11
12	9 (+)	60 (+)	90 (+)	30 (−)	30.38	30.31	0.00	7.98	7.79	0.02	22.72	23.93	0.05	5.89	6.69	0.12
13	9 (+)	60 (+)	10 (−)	30 (−)	21.48	21.65	0.01	5.63	5.56	0.01	21.04	20.19	0.04	5.47	5.72	0.04
14	6 (0)	35 (0)	50 (0)	75 (0)	30.73	28.91	0.06	1.86	1.75	0.06	15.79	15.27	0.03	0.96	0.94	0.02
15	3 (−)	60 (+)	10 (−)	120 (+)	38.57	40.25	0.04	2.51	2.66	0.06	29.28	28.81	0.02	1.90	1.83	0.04
16	9 (+)	60 (+)	90 (+)	120 (+)	59.51	63.10	0.06	3.82	4.06	0.06	49.65	53.60	0.07	3.22	3.67	0.12
17	9 (+)	10 (−)	90 (+)	30 (−)	36.33	32.83	0.11	1.21	1.33	0.09	44.49	40.97	0.09	1.92	1.81	0.06
18	3 (-)	10 (−)	90 (+)	120 (+)	57.29	61.02	0.06	0.62	0.64	0.01	61.39	58.49	0.05	0.66	0.58	0.14
19	9 (+)	10 (−)	10 (−)	120 (+)	54.98	48.81	0.13	0.52	0.50	0.05	81.27	77.43	0.05	0.88	0.85	0.04
20	6 (0)	35 (0)	50 (0)	75 (0)	29.36	28.91	0.02	1.78	1.75	0.02	15.11	15.27	0.01	0.92	0.94	0.02
21	6 (0)	35 (0)	50 (0)	75 (0)	32.12	28.91	0.11	1.95	1.75	0.11	16.36	15.27	0.07	0.99	0.94	0.06
22	6 (0)	35 (0)	50 (0)	75 (0)	29.32	28.91	0.01	1.78	1.75	0.02	16.06	15.27	0.05	0.97	0.94	0.04
23	6 (0)	35 (0)	50 (0)	75 (0)	26.09	28.91	0.10	1.58	1.75	0.10	14.42	15.27	0.06	0.87	0.94	0.07
24	6 (0)	35 (0)	50 (0)	75 (0)	26.82	28.91	0.10	1.62	1.75	0.08	13.81	15.27	0.01	0.84	0.94	0.10
25	9 (+)	10 (−)	90 (+)	120 (+)	70.03	68.34	0.02	0.77	0.69	0.11	92.99	91.78	0.01	1.01	1.00	0.02
26	3 (−)	60 (+)	90 (+)	30 (−)	29.27	27.07	0.10	7.59	7.14	0.06	16.18	15.25	0.06	4.18	3.89	0.07
27	3 (−)	10 (−)	10 (−)	30 (−)	18.36	20.94	0.12	0.79	0.87	0.02	21.61	22.03	0.04	0.93	0.90	0.10
28	9 (+)	60 (+)	10 (−)	30 (−)	21.48	21.65	0.01	5.64	5.56	0.04	21.42	20.19	0.01	5.55	5.72	0.01

* Obs: observed values; ** Pred: predicted values; *** RE = ǀ (True value—Observed value)ǀ/True value.

**Table 3:** Brunauer-Emmett-Teller (BET) analysis of the SCBC and TiO_2_@SCBC.

Parameters	SCBC	TiO_2_@SCBC
Langmuir surface area (SA) (m^2^/g)	49.23	50.54
Total pore volume (cm^3^/g)	0.058	0.048
Average pore radius (°A)	30.3	24.4

**Table 4:** Analysis of Variance (ANOVA) testing output.

SCBC										
Response	%R					*q_e_*				
Source	DF *	Adj SS *	Adj MS *	*F*-Value	*p*-Value	DF *	Adj SS *	Adj MS *	*F*-Value	*p*-Value
Model	5	3.64890	0.72978	125.67	0.000	5	18.3315	3.6663	842.07	0.000
Linear	4	3.34975	0.83744	144.21	0.000	4	18.3022	4.5756	1050.90	0.000
pH	1	0.06412	0.06412	11.04	0.003	1	0.0372	0.0372	8.54	0.008
[BALX]	1	0.03176	0.03176	5.47	0.029	1	15.5721	15.5721	3576.55	0.000
CT	1	0.56584	0.56584	97.44	0.000	1	0.5694	0.5694	130.79	0.000
AD	1	2.68803	2.68803	462.88	0.000	1	2.1236	2.1236	487.73	0.000
Curvature	1	0.29915	0.29915	51.51	0.000	1	0.0293	0.0293	6.73	0.017
Error	22	0.12776	0.00581			22	0.0958	0.0044		
Lack-of-Fit	10	0.06322	0.00632	1.18	0.390	10	0.0294	0.0029	0.53	0.838
Pure Error	12	0.06454	0.00538			12	0.0664	0.0055		
Total	27	3.77666				27	18.4273			
**TiO_2_@SCBC**										
**Response**	**%R**					***q_e_***				
Source	DF *	Adj SS *	Adj MS *	*F*-Value	*p*-Value	DF *	Adj SS *	Adj MS *	*F*-Value	*p*-Value
Model	5	9.61640	1.92328	543.22	0.000	5	14.4461	2.88922	1482.04	0.000
Linear	4	5.85847	1.46462	413.67	0.000	4	11.9301	2.98252	1529.90	0.000
pH	1	1.01483	1.01483	286.63	0.000	1	1.4524	1.45244	745.03	0.000
[BALX]	1	1.44657	1.44657	408.58	0.000	1	8.5523	8.55228	4386.92	0.000
CT	1	0.14464	0.14464	40.85	0.000	1	0.1206	0.12056	61.84	0.000
AD	1	3.25243	3.25243	918.63	0.000	1	1.8048	1.80482	925.79	0.000
Curvature	1	3.75793	3.75793	1061.40	0.000	1	2.5160	2.51602	1290.60	0.000
Error	1	3.75793	3.75793	1061.40	0.000	1	2.5160	2.51602	1290.60	0.000
Lack-of-Fit	22	0.07789	0.00354	1.85	0.155	22	0.0429	0.00195	1.29	0.335
Pure Error	10	0.04729	0.00473			10	0.0222	0.00222		
Total	12	0.03060	0.00255			12	0.0207	0.00173		

* DF is degrees of freedom, SS is sum of squares, and MS is mean of squares.

**Table 5:** Regression models and the assessment parameters, and the optimum conditions.

Response	R^2^%	R^2^–Adj %	R^2^–Pred %	Optimum Conditions
%R _(SCBC)_	96.62	95.85	94.46	pH = 9.0, [BALX] = 10 ppm, CT = 90 min, AD = 120 mg, (*d* = 0.9672, %R = 68.34%)
*q_e_*_(SCBC)_	99.48	99.36	99.17	pH = 9.0, [BALX] = 60 ppm, CT = 90 min, AD = 30 mg, (*d* = 0.9740, *q_e_* = 7.79 mg/g)
%R _(TiO2@SCBC)_	99.20	99.01	98.70	pH = 9.0, [BALX] = 10 ppm, CT = 90 min, AD = 120 mg, (*d* = 0.9848, %R = 91.78%)
*q_e_*_(TiO2@SCBC)_	99.70	99.64	99.52	pH = 9.0, [BALX] = 60 ppm, CT = 90 min, AD = 30 mg, (*d* = 1.000, *q_e_* = 6.69 mg/g)

**Table 6:** Nonlinear equations of Langmuir, Freundlich, Temkin and D-R isotherms, beside their parameters for the adsorption of BALX on both SCBC and TiO_2_@SCBC.

Isotherm	Equations (Nonlinear Forms)	Parameters	Value	
			SCBC	TiO_2_@SCBC
Langmuir	$q_{e}=\frac{q_{m}\;K_{L}\;C_{eq}}{1+K_{L}\;C_{eq}}$	$q_{m}$ (mg/g)	142.55	196.73
		$K_{L}$ (L. mole^−1^)	0.031	0.019
		R^2^	0.9803	0.9784
Freundlich	$q_{e}=K_{F}C_{eq}^{\frac{1}{n}}$	1n	0.78	0.84
		$K_{F}$ (mole/g) (L/mole)^1/n^	5.47	4.45
		R^2^	0.9844	0.9831
Temkin	$q_{e}=\frac{RT}{b_{T}}\;\ln\left(A_{T}\;C_{eq}\right)$	$b_{T}\;$(J/mole)	221.6	224.7
		$A_{T}$ (L/mole)	1.992	1.753
		R^2^	0.8867	0.8645
D-R	$q_{e}=q_{s\;}\;$. exp (−β.$\;\epsilon^{2}$)	β	1.04 × 10^−8^	9.97 × 10^−9^
	$\epsilon =RT\left(1+\frac{1}{C_{eq}}\right)$	E (kJ/mole)	6.93	7.08
		$q_{m}$ (mg/g)	50.29	47.17
	E=12β	R^2^	0.8732	0.8747

**Table 7:** Kinetics of the adsorption of BALX onto SCBC and TiO_2_@SCBC.

Model	Parameter	Value	
		SCBC	TiO_2_@SCBC
PFO $\frac{dq_{t}}{dt}$ *= k*_1_*(q_e −_ q_t_)*	*K*_1_ (min^−1^)	1.826	1.263
	*q_e_* (mg/g)	38.48	38.98
	R^2^	0.4884	0.6899
PSO $\frac{dq_{t}}{dt}$ = *k*_2_*(q_e_ − q_t_)*^2^	*K*_2_ (g.mg^−1^ min^−1^)	0.113	0.064
	*q_e_* (mg/g)	39.23	40.04
	R^2^	0.8705	0.9451
Elovich *q_t_=* $\frac{1}{\beta }\times ln\left(1+\alpha \beta t\right)$	*α*	2.44 × 10^11^	1.18 × 10^8^
	*B*	0.759	0.549
	R^2^	0.9135	0.7694
Weber-Morris $q_{t}=K_{I}t^{0.5}+C$	*K_I_*	0.509	0.649
	*C*	35.32	34.63
	R^2^	0.6863	0.4910

Where *K*_2_ is rate constant (g mg^−1^ min^−1^) and *q_t_* is adsorbed quantity at time *t*; while *α* and *β* are initial sorption concentration rate (mg g^−1^ min^−1^), and desorption constant (g/mg), *K_I_* is intraparticle diffusion rate constant (mg g^−1^ min^−0.5^), and *C* is boundary thickness effect.

## Data Availability

The data presented in this study are available within this article. Further inquiries could be directed to the authors.

## Supplementary Materials

The following are available online. Table S1: A comparison between different adsorbents derived from coffee grounds and husks for the removal of pharmaceuticals.

## Sample Availability

Not available.
