# Supplementary material for: Biochar of Spent Coffee Grounds as Per Se and Impregnated with TiO2: Promising Waste-Derived Adsorbents for Balofloxacin
Source: Molecules. 2021 Apr 15;26(8):2295. doi: 10.3390/molecules26082295 (PMC8071459; doi:10.3390/molecules26082295)
Supplement: Supplementary file 1 [file molecules-26-02295-s001.zip › molecules-1181798-supplementary.pdf]

Biochar of Spent Coffee Grounds as Per Se and Impregnated with TiO_2: Promising Waste–Derived Adsorbents for Balofloxacin

Marwa El-Azazy *, Ahmed S. El-Shafie, and Hagar Morsy

Department of Chemistry and Earth Sciences, College of Arts and Sciences, Qatar University, Doha 2713, Qatar; marwasaid@qu.edu.qa (M.E.-A); aelshafie@qu.edu.qa (A.S.E.-S.); hm1604839@student.qu.edu.qa (H.M.)

Table S1. A comparison between different adsorbents derived from coffee grounds and husks for the removal of pharmaceuticals.

Adsorbent	Adsorbate	Analytical Approach	Kinetic Model	Isotherm Model	Surface Area (m^2/g)	q_{max} (mg/g)	%R	Ref
Pristine Spent coffee grounds (SCBC), Impregnated with titanium oxide (TiO_2@SCBC)	Balofloxacin (BALX)	Batch adsorption, Plackett–Burman design (PBD)	*PSO	Freundlich	49.23 (SCBC)<br>50.54 (TiO_2@SCBC)	142.55 (SCBC)<br>196.73 (TiO_2@SCBC)	68.34% (SCBC)<br>91.78% (TiO_2@SCBC)	This work
Chitosan/waste coffee-grounds composites	Metamizol (MET),<br>Acetylsalicylic acid (ASA)<br>Acetaminophen (ACE)<br>Caffeine (CAF)	Batch adsorption, univariate analysis	*PSO	Freundlich	NS	NS	Variable (ASA > CAF > ACE > MET)	[29]
Pristine and NaOH-activated biochars from spent coffee wastes (SCW)	Naproxen (NPX)<br>Diclofenac (DCF)<br>Ibuprofen (IBU)	Batch adsorption, univariate analysis	*PSO	Freundlich (pristine) Langmuir (NaOH-activated)	62.0 (pristine)<br>655.4 (NaOH-activated)	[1]NPX 107.53<br>[1]DCF 91.74<br>[1]IBU 86.21<br>[2]NPX 344.48<br>[2]DCF 202.92<br>[2]IBU 124.14<br>[3]NPX 269.01<br>[3]DCF 97.17<br>[3]IBU 76.10<br>[4]NPX 263.34<br>[4]DCF 97.12<br>[4]IBU 74.07	NS	[30]
Coffee husk (CH), using NaOH or $ZnCl_2$ as activating agents	Acetaminophen (ACE)	Batch adsorption, univariate analysis	*PSO	Redlich-Peterson	1 (CH)<br>613 (NaOH-activated)<br>620 ($ZnCl_2$-activated)	50.25 (distilled water)<br>48.31 (urine)	> 95%	[31]
Natural state (NAC), thermally activated at 450 °C (TAC) and chemically activated in the presence of	Paracetamol (PCM)	Batch adsorption, univariate analysis	*PSO	Freundlich	102.2 (NAC)<br>648.6 (TAC) 888.1 (CAC)	NS	98%	[32]

phosphoric acid (CAC)					

*PSO: Pseudo-second order, [1,3] Pristine SCW biochar in lake water, [2,4] NaOH-activated SCW biochar, [1,2,3,4] q_{max} = µmol/g
